# Using digital soil maps to infer edaphic affinities of plant species in Amazonia: Problems and prospects

**DOI:** 10.1002/ece3.3242

**Published:** 2017-09-12

**Authors:** Gabriel Massaine Moulatlet, Gabriela Zuquim, Fernando Oliveira Gouvêa Figueiredo, Samuli Lehtonen, Thaise Emilio, Kalle Ruokolainen, Hanna Tuomisto

**Affiliations:** ^1^ Department of Biology University of Turku Turku Finland; ^2^ Programa de Pesquisas em Biodiversidade – PPBio Instituto Nacional de Pesquisas da Amazônia ‐ INPA Manaus AM Brazil; ^3^ Programa de Pós‐Graduação em Ecologia Instituto Nacional de Pesquisas da Amazônia ‐ INPA Manaus AM Brazil; ^4^ Biodiversity Unit University of Turku Turku Finland; ^5^ Comparative Plant and Fungal Biology Royal Botanic Gardens Richmond London UK; ^6^ Department of Geography and Geology University of Turku Turku Finland

**Keywords:** Amazonia, ferns, HWSD, soil properties, SoilGrids, SOTERLAC, species distribution modeling, species tolerances

## Abstract

Amazonia combines semi‐continental size with difficult access, so both current ranges of species and their ability to cope with environmental change have to be inferred from sparse field data. Although efficient techniques for modeling species distributions on the basis of a small number of species occurrences exist, their success depends on the availability of relevant environmental data layers. Soil data are important in this context, because soil properties have been found to determine plant occurrence patterns in Amazonian lowlands at all spatial scales. Here we evaluate the potential for this purpose of three digital soil maps that are freely available online: SOTERLAC, HWSD, and SoilGrids. We first tested how well they reflect local soil cation concentration as documented with 1,500 widely distributed soil samples. We found that measured soil cation concentration differed by up to two orders of magnitude between sites mapped into the same soil class. The best map‐based predictor of local soil cation concentration was obtained with a regression model combining soil classes from HWSD with cation exchange capacity (CEC) from SoilGrids. Next, we evaluated to what degree the known edaphic affinities of thirteen plant species (as documented with field data from 1,200 of the soil sample sites) can be inferred from the soil maps. The species segregated clearly along the soil cation concentration gradient in the field, but only partially along the model‐estimated cation concentration gradient, and hardly at all along the mapped CEC gradient. The main problems reducing the predictive ability of the soil maps were insufficient spatial resolution and/or georeferencing errors combined with thematic inaccuracy and absence of the most relevant edaphic variables. Addressing these problems would provide better models of the edaphic environment for ecological studies in Amazonia.

## 1 INTRODUCTION

Information on habitat preferences of species is important to understand biogeography and macroecology, and to make justified conservation decisions and land use planning (Margules & Pressey, 2000). Amazonia is the world's largest tropical rainforest and an important repository of species diversity, but it is still poorly explored by researchers (Feeley, 2015; ter Steege et al., 2016; Zappi et al., 2015). Recently, climate change has raised concerns about species tolerances to the changing environment and possible shifts in species distributions (Feeley & Silman, 2016). Mapping suitable habitats for species with different habitat requirements would help to delimit a network of strategically placed conservation units that collectively represent the heterogeneity within the biome. However, a major practical problem is that field observations for biotic and abiotic data available for species distribution modeling are scanty and geographically biased (McMichael, Matthews‐Bird, Farfan‐Rios, & Feeley, 2017).

Recent advances in Geographic Information Systems (GIS), statistical techniques, and in the availability of biodiversity and environmental databases have inspired a rapid development in the modeling of species distributions (Barbosa & Schneck, 2015). Species distribution models (SDMs) in data‐rich continents and ecosystems can take advantage of a broad range of environmental variables and large numbers of species records (Mod, Scherrer, Luoto, & Guisan, 2016). At the same time, semi‐continental areas such as Amazonia suffer simultaneously from poor species data coverage, which would make SDMs especially important, and from limited availability and poor accuracy of environmental data layers, which renders the results of such analyses less reliable.

Climatic layers have been the most widely used variables in broad‐scale SDMs both because climatic factors are an important environmental determinant of species ranges (Feeley, 2012) and because climatic data are readily available in digital format (e.g., WorldClim; Hijmans, Cameron, Parra, Jones, & Jarvis, 2005). Variation in rainfall seasonality indeed seems to affect species distributions in Amazonia (Esquivel‐Muelbert et al., 2016; ter Steege et al., 2006; Toledo et al., 2011). However, climatic variation is unlikely to be the only (or even the main) cause of compositional variation, especially in the central parts of Amazonia, where climate is most humid and least seasonal. Several studies have indeed found soil factors to be of greater importance than climatic factors in shaping plant communities in Amazonia (ter Steege et al., 2006; Tuomisto & Poulsen, 1996; Tuomisto, Zuquim, & Cárdenas, 2014; Zuquim et al., 2012). In particular, the concentration of base cations in the soil (Ca, Mg, K, and Na) has been strongly linked to floristic variation across the lowlands (Higgins et al., 2011; Phillips et al., 2003; Pitman et al., 2008; Tuomisto, Ruokolainen, Aguilar, & Sarmiento, 2003; Tuomisto et al., 2016). It has also been suggested that niche partitioning along the soil cation concentration gradient is a mechanism that promotes speciation and regional coexistence of closely related species (Fine, Daly, & Cameron, 2005; Tuomisto, 2006).

In spite of their physiological importance and proven relationships with plant distributions, edaphic variables have rarely been used in SDMs. This may be either due to the low resolution and accuracy of the available soil maps or the generally held idea that soils are only relevant at the local scale (Coudun, Gégout, Piedallu, & Rameau, 2006; Grunwald, Thompson, & Boettinger, 2011). However, edaphic variables have recently been shown to improve the explanatory power of SDMs across European landscapes (Bertrand, Perez, & Gégout, 2012; Dubuis et al., 2013). In Amazonia, the need of digital soil maps and other edaphic GIS layers has intensified due to rapid environmental changes and the concern about the current status of soil resources and the biodiversity associated with them (Grunwald et al., 2011; Laurance et al., 2002). Increasing understanding of the tight relationship between plant species occurrences and soil properties also motivates the use of edaphic GIS layers for predicting the distributions of plant species. Indeed, a recent study made inferences about the relative importance of past human influences and current environmental effects on the distribution patterns of Amazonian trees using Cation Exchange Capacity (CEC) values obtained from a digital soil map (Levis et al., 2017). The main challenge is that soil properties can vary considerably over small distances and depths (Lips & Duivenvoorden, 1996; Luizão et al., 2004; Quesada et al., 2011), and the procedures to interpolate between scanty primary soil data localities might produce maps whose accuracy is low at the scales that are relevant for the study at hand.

Amazon‐wide soil maps are currently available digitally. Three of them have been used in species diversity assessments. The global Soil and Terrain Database (SOTER) is a well‐known polygon‐based map. The version for Latin America and the Caribbean (SOTERLAC; Dijkshoorn, Huting, & Tempel, 2005) is a compilation of soil information that has been put together over several decades and it provides a soil map with a minimum map scale of 1:1 million. The Harmonized World Soil Database (HWSD; Nachtergaele, Velthuizen, Verelst, & Wiberg, 2012) provides a raster map with 1‐km spatial resolution. It is based on the same data as SOTER but includes also information from national soil databases. Rather than classifying each pixel to a single soil type, HWSD provides a coverage probability for each soil class in each pixel. Another raster map is SoilGrids (Hengl et al., 2014, 2017), which has a 250‐m spatial resolution and provides chemical and physical soil variables in addition to occurrence probabilities for soil classes. The SoilGrids information is derived from statistical modeling of soil properties, and the interpolation between actual soil profiles was done using machine learning.

Recently, digital soil layers have started to be used for modeling different aspects of biodiversity in the Neotropics (Albuquerque & Beier, 2015; Kissling et al., 2012; Levis et al., 2017; McMichael, Palace, & Golightly, 2014; McPherson, 2014; Poorter et al., 2015; Thomas, Alcázar Caicedo, Loo, & Kindt, 2014). In these studies, either the number of soil classes was used as an indicator of habitat heterogeneity or soil CEC was extracted from the maps and used as an explanatory variable in data analyses. However, validation of digital soil maps depends on the availability of local soil information, so the thematic accuracy of the information that the maps provide for poorly sampled areas such as Amazonia may be low when compared to other parts of the globe (Grunwald et al., 2011; Hengl et al., 2014, 2017; Sollins, 1998). This raises the question: How well will the predictions of species occurrences based on soil maps reflect the actual associations between species and soil properties? The inherent accuracy issues of soil maps have been discussed elsewhere (Brevik et al., 2016; Grunwald et al., 2011; Hartemink, Krasilnikov, & Bockheim, 2013; Palm, Sanchez, Ahamed, & Awiti, 2007), so here we focus on those aspects that are most relevant when using digital soil maps to infer species edaphic niches.

Evaluating to what degree species niches may be reconstructed incorrectly due to problems in environmental data layers requires species data that combine standardized taxonomy with field‐measured environmental data, and such data are sparse (ter Steege et al., 2016). Here we use a dataset on fern species occurrences and soil cation concentration that has both broad geographic coverage and high taxonomical consistency. We use these data to determine edaphic preferences of thirteen plant species using both field data and information extracted from the three digital soil maps. We then test the correspondence between the results obtained with the different data sources. We specifically ask (1) if soil classes mapped in SOTERLAC, HWSD, and SoilGrids can be used as surrogates of local soil cation concentration within the Amazon rain forest biome; (2) how well the information extracted from digital soil maps reflects species edaphic affinities as inferred from field data; and (3) what are the current caveats when using digital soil maps to determine plant species niches across Amazonia.

## 2 METHODS

### 2.1 Digital soil data

We used data from three digital soil maps in our analyses: SOTERLAC, HWDS, and SoilGrids. The SOTERLAC v2.0 soil map was downloaded from the FAO‐ISRIC webpage (http://geonode.isric.org/layers/geonode:soter_lac_map_unit, downloaded in December 2016). The minimum map scale is 1:1 million for Brazil and Peru and 1:5 million for the rest of Latin America. SOTERLAC uses soil classes, topology, and terrain characteristics to delineate polygons, having the Digital Chart of the World as a cartographic base. Each polygon has a soil class attribute (e.g., Haplic Acrisols) as defined by the World Reference Base for soil resources (FAO 2006).

The Harmonized World Soil Database v1.1 (HWSD) is composed of a set of layers that we downloaded from Worldgrids portal of the ISRIC‐World Soil Information (http://www.worldgrids.org/doku.php?id=wiki:layers, downloaded in December 2016). Each of the 30 layers corresponds to one of the WRB‐FAO dominant soil classes, with the pixel values expressing its probability of occurrence at a resolution of 30 arc‐seconds (ca. 1 km at the Equator). HWSD scale is 1:5 million, and it uses harmonized soil classes and soil properties combined from national and regional databases. The grid cells provide the same attributes as the original soil maps used for the harmonization (Nachtergaele et al., 2012).

SoilGrids has two versions, one at 1‐km resolution and the other at 250‐m resolution. We used the 250‐m data, which is hereafter simply referred to as SoilGrids (Hengl et al., 2014, 2017). SoilGrids is a pixel‐based map composed of a set of layers in raster format that contain either information related to the soil classification or to specific physical and chemical properties. The layers with data on the WRB‐FAO soil classes (layers coded as TAXNWRB) were downloaded from the SoilGrids portal (http://soilgrids.org, downloaded in December 2016). As with HWSD, each soil class is stored as a separate layer and each pixel has a value corresponding to the probability of occurrence of that soil class. SoilGrids was produced by machine learning algorithms and it used 158 covariates as model input.

The soil class attribute of the SOTERLAC polygons is based on a more detailed soil classification scheme than the HWSD dominant soil class data and SoilGrids soil classes. To allow comparison among the datasets, we added to each SOTERLAC polygon a new soil class attribute based on the WRB‐FAO dominant soil classes. This was obtained by applying the aggregation of soil classes proposed by Quesada et al. (Quesada et al., 2011). The soil classes and acronyms that are relevant to this study are listed in the Appendix 1, Table A1.

None of the three soil maps contains information on the concentration of exchangeable base cations (Ca, Mg, and K) for Amazonia, but SoilGrids provides a layer with data on cation exchange capacity (CEC, in cmol(+)/kg). The concentration of exchangeable bases is a quantitative measure of the availability of these nutrient cations in the soil. In contrast, CEC measures the overall potential of the soil to exchange cations, including the acid aluminum, which is not a plant nutrient. Out of the CEC layers that are available in SoilGrids, we downloaded CEC values as estimated for the top 5 cm of soil (layer CECSOL_M_sl2_250m_l1), as also our field data were based on surface soil samples.

### 2.2 Field data

We carried out fieldwork in non‐inundated (terra firme) forests in lowland (<400 m elevation) Amazonia in the context of two originally independent research programs. Most of the western Amazonian data were collected by the Amazon Research Team of the University of Turku (hereafter referred to as UTU), and most of the central Amazonian data by the Brazilian Program of Biodiversity Research (hereafter referred to as PPBio). Within each program, soil sampling and quantitative fern inventories were done using plots of a fixed surface area, but the length, shape, and topographical orientation of the plots differed between programs. All plots were georeferenced using coordinates taken with a handheld GPS in the field.

The PPBio inventories included 326 permanent plots of 250 m × 2 m. These were established along the terrain isoclines in order to minimize local soil heterogeneity (Magnusson et al., 2005). In each plot, six surface soil samples (the top 5 cm of the mineral soil) were taken at every 50 m and bulked to obtain a single composite sample. The soil samples were analyzed for exchangeable Ca, K, Mg in the Plant and Soil Thematic Laboratory of Brazilian National Institute for Amazonian Research (LTSP‐INPA) using the Mehlich I protocol (KCl 1N method; Donagena, Campos, Calderano, Teixera, & Viana, 1997). For simplicity, the concentration of Ca, Mg, and K as expressed in cmol(+)/kg will henceforth be referred to as soil cation concentration. PPBio data are available at https://ppbio.inpa.gov.br/repositorio/dados.

The UTU inventories included 311 temporary line transects that were 5 m wide and either 500 m or 1,300 m long. The transects were generally perpendicular to terrain isoclines in order to maximize local soil heterogeneity (Tuomisto et al., 2003). Composite surface soil samples (top 5 cm of the mineral soil) were taken at about 200‐m intervals such that they represented the topographical extremes within the transect. Each soil sample consisted of five subsamples collected within an area of about 5 m by 5 m and bulked. For the purposes of the present paper, we extracted 150‐m‐long segments from the UTU transects. Each of these 879 plots contains exactly one composite soil sample, and if adjacent plots would have overlapped, one of them was excluded. This improves the accuracy of the soil data in relation to the plant occurrence data. The soil samples were analyzed for soil cation concentration at MTT Agrifood Research (Jokioinen, Finland) using extraction in 1 M ammonium acetate (van Reeuwijk, 1993). Although concentration of Na was analyzed for the UTU samples, it is not used here, because it was not available for the PPBio samples.

**Figure 1:**
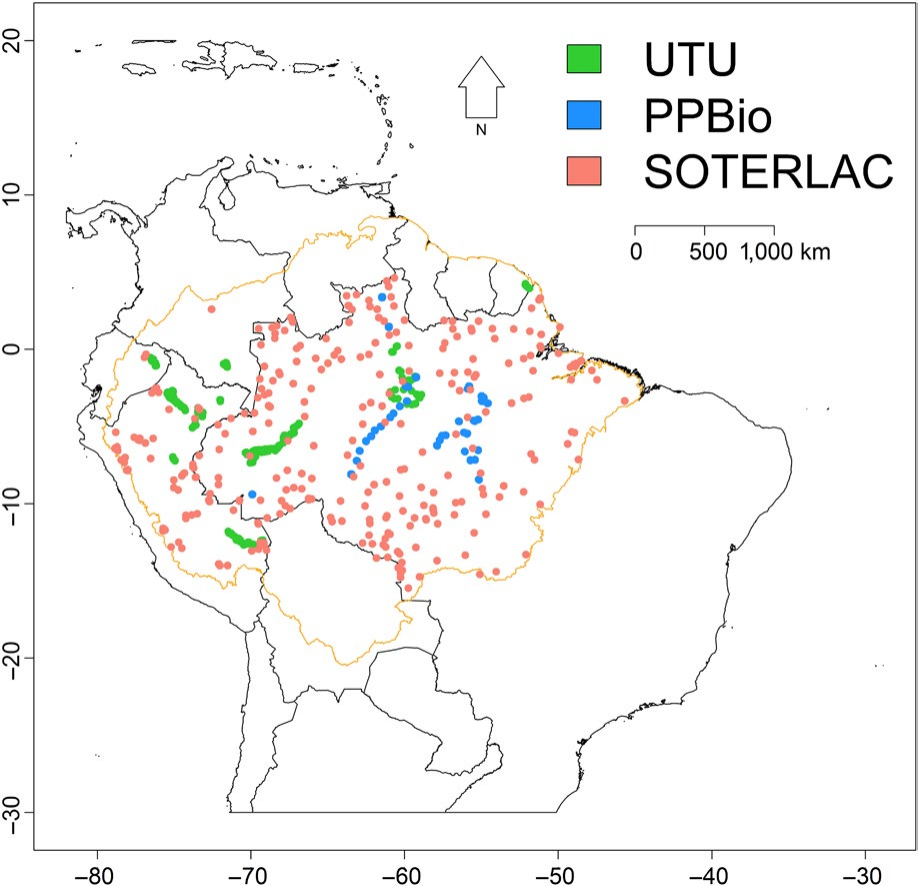
Distribution of the 1505 surface soil samples used in this study (879 samples from UTU, 326 from PPBio, and 300 from the SOTERLAC database (Batjes, 2005). Limits of Amazonia are indicated by the orange line (Eva & Huber, 2005)

In addition, we used published data on soil cation concentration associated with the SOTERLAC database v2.0 (Batjes, 2005; Dijkshoorn et al., 2005; hereafter referred to as SOTERLAC) from 300 soil profiles across Amazonia. Some of the available data concerned deeper soil horizons, but we only used soil samples taken within the topmost 30 cm. The laboratories and procedures used to analyze the SOTERLAC soil samples are listed in the SOTERLAC metadata. The spatial distributions of the data points obtained from the three soil datasets (UTU, PPBio, and SOTERLAC) are shown in Figure 1. In general, nutrient stocks in Amazonian soils are higher in the top 5 cm than in deeper soil horizons (Johnson, Vieira, Zarin, Frizano, & Johnson, 2001; Quesada et al., 2011), so it is possible that the SOTERLAC soil samples give slightly smaller cation concentrations for similar soils than the UTU and PPBio samples, but we do not expect this to significantly bias the analyses.

In addition to soil data, both UTU and PPBio plots provided data on plant species occurrences. Here we focus on thirteen fern taxa that fulfill the following criteria: 1) They were well represented in both datasets; 2) earlier studies have found them to be indicators of specific parts of the soil cation concentration gradient (Tuomisto & Poulsen, 1996; Tuomisto, Ruokolainen et al., 2003; Zuquim et al., 2014); 3) they collectively span that gradient; and 4) they are easy to identify, which makes it possible to combine the PPBio and UTU data without having cross‐checked voucher specimens. The selected species were as follows: *Adiantum pulverulentum*,* Adiantum tomentosum*,* Cyathea pungens*,* Cyclopeltis semicordata*,* Lindsaea guianensis*,* Pteris pungens*,* Saccoloma inaequale*,* Schizaea elegans*,* Thelypteris macrophylla*,* Trichomanes elegans,* and *Trichomanes martiusii*. In addition, we included *Metaxya* and *Triplophyllum* at the generic level: Each has only a few closely related species that have similar distributions along the soil cation concentration gradient and are morphologically so similar that they can easily be confused in the field. In each plot, all terrestrial fern individuals were recorded that had at least one leaf longer than a predefined minimum (5 cm for PPBio [but see (Zuquim et al., 2012) for exceptions], 10 cm for UTU).

### 2.3 Correspondence between soil classes and local soil data

Because soil cation concentration has consistently emerged as a good predictor of plant species occurrence patterns, we first assessed if the mapped soil class information that is available in SOTERLAC corresponds with the soil cation concentration values measured in the soil samples of UTU, PPBio, and SOTERLAC. Each soil sample was assigned to a soil class on the basis of its coordinates. This allowed both assessing the variability within the mapped soil classes and testing for differences in mean soil cation concentration between them. The latter was done using ANOVA followed by Tukey's test.

We used multiple linear regression models to evaluate how well local soil cation concentration can be predicted using the soil class probabilities of HWSD and SoilGrids, and the CEC values of SoilGrids. We built separate models for HWSD and SoilGrids, with and without CEC. Soil cation concentrations obtained from UTU, PPBio, and SOTERLAC field samples were the response variable, and initial model configuration had as explanatory variables all the downloaded soil classes. The significant variables of each model were selected by a stepwise forward–backward procedure. We identified the best model with the lowest Akaike information criterion (AIC). We used the predicted soil cation concentrations from these models to reconstruct species–soil associations. These analyses were carried out separately for the UTU, PPBio, and SOTERLAC soil data, as well as all three soil datasets combined.

### 2.4 Correspondence of the geographic limits of soil classes with landscape features

As the SOTERLAC map is based on polygons, the soil classes are clearly defined by borders. Although the HWSD is a pixel map, soil class probabilities in them reflect broader patterns similar to those in SOTERLAC. This makes it possible to check if such landscape features that are typically associated with specific soil types actually match the mapped distribution of those soil types. We focused on the contrast between non‐inundated (terra firme) areas and the floodplains of major rivers, because the limit between the two is readily identifiable in SRTM (Shuttle Radar Topography Mission) data, and floodplains typically have such soil types that rarely occur in terra firme (e.g., Gleysols and Fluvisols). We used ArcGIS v10.1 to overlay the soil maps and SRTM. Then, we visually scanned through the Amazon basin to assess how well the floodplain‐associated soil classes matched the extent of the floodplains as interpreted from SRTM. All data layers used the same datum (WGS84) and projection (Lat/Long).

### 2.5 Species affinities to soil properties

To estimate where the abundance of each taxon peaks along the soil cation concentration gradient, we calculated the soil cation concentration optimum for every taxon (sensu ter Braak & van Dam, 1989). This equals the weighted average of the soil cation concentration values in those plots where the taxon occurred, with the taxon's abundance used as the weight (eq. 4 in ter Braak & van Dam, 1989). In addition, we calculated a tolerance for each taxon as the root mean squared error (RMSE) around the optimum. This was done separately for the soil cation concentration values that had been measured from field samples and those values that were predicted with multiple regression models on the basis of HWSD and SoilGrids. For comparison, we also calculated optima and tolerances for CEC as extracted from SoilGrids. The rank correlation between the field‐based and model‐based optima was quantified using Kendall's tau.

All data analyses were performed in R using code written by GMM and the packages *vegan* (Oksanen et al., 2015), *rioja* (Juggins, 2015), *ggplot2* (Wickham, Chang, & Wickham, 2013), *dplyr* (Wickham & Francois, 2016), *maptools* (Bivand & Lewin‐Koh, 2016), and *rgdal* (Bivand, Keitt, & Rowlingson, 2016).

## 3 RESULTS

### 3.1 Soil cation concentration and mapped soil classes

The SOTERLAC soil dataset covered Amazonia more evenly than the other datasets did (Figure 1), and the majority of the soil classes in the SOTERLAC soil map were represented by at least one SOTERLAC soil profile. In contrast, less than half of the SOTERLAC soil classes were represented in the UTU and PPBio soil datasets (Figure 1). For example, soils that are typically found along rivers, such as Fluvisols and Gleysols, were absent in the PPBio dataset because the PPBio sampling was concentrated in interfluvial areas.

**Figure 2:**
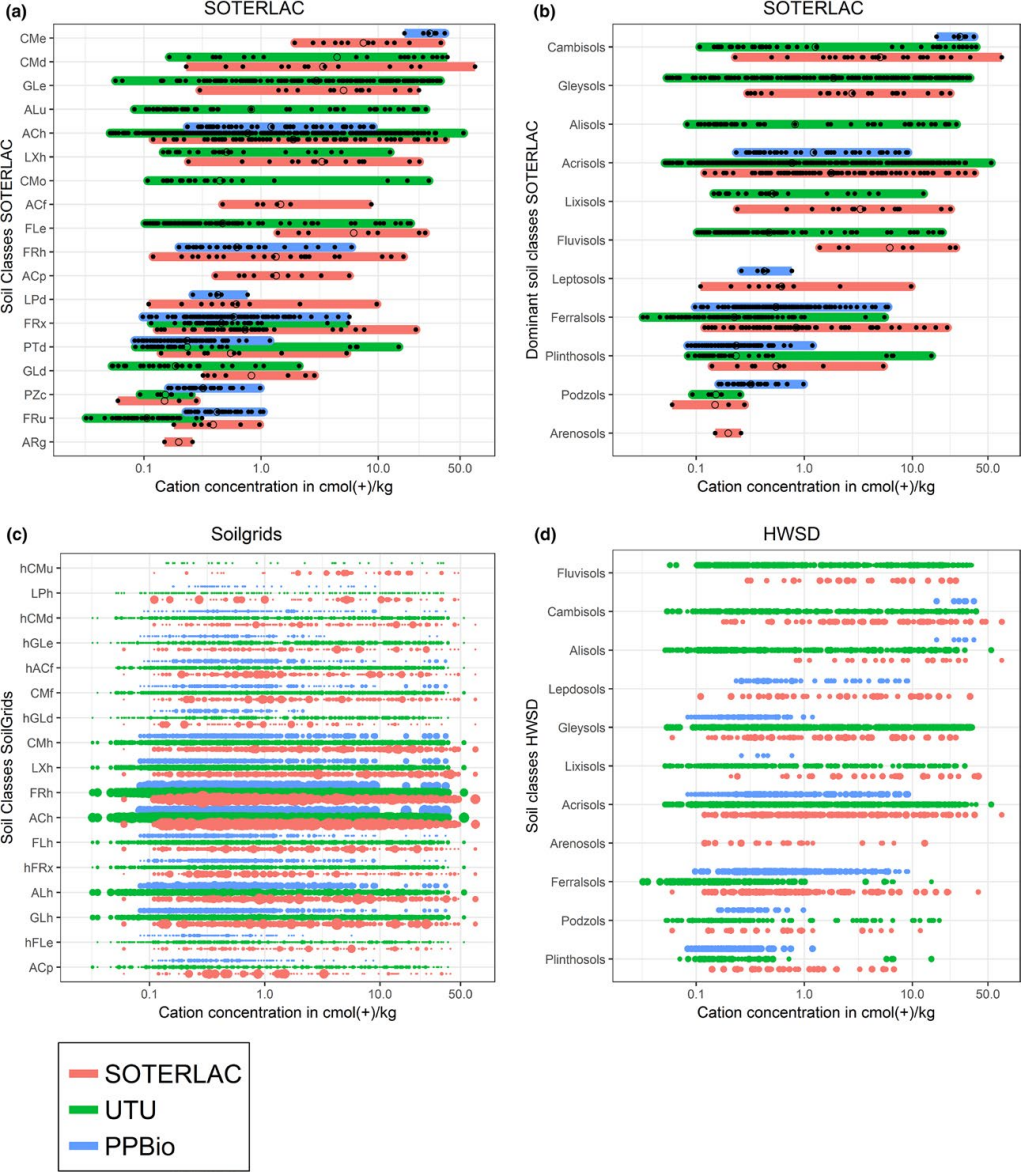
The distribution of soil cation concentrations as measured in soil samples of three different datasets (SOTERLAC, UTU, and PPBio) within soil classes as represented in three digital soil maps of Amazonia (SOTERLAC, SoilGrids, and HWSD). In (a) and (b), the colored lines indicate the total range of cation concentration values, the small black dots the values measured in individual soil samples, and the open circles the corresponding means. In (c) and (d), each colored dot corresponds to a soil sample, and dot size is proportional to the probability that the corresponding pixel in the digital soil map contains the indicated soil class. Only soil classes that were represented in UTU and/or PPBio data are shown. Soil classes are ordered by their mean cation concentration value as calculated using all soil sample data. For explanations of the soil class acronyms in (a) and (c), see Appendix 1, Table A1

Almost all SOTERLAC soil classes had broad ranges of soil cation concentration, and soil samples assigned to the same soil class could differ in cation concentration by up to two orders of magnitude (Figure 2, Table A1). Nevertheless, soil classes with the highest soil cation concentration values were significantly different from those with the lowest values (Table 1). The correlation between field‐measured soil cation concentration and CEC from SoilGrids was statistically significant but weak (Pearson's *r* = 0.106, *p* < .001). The explanatory power (adjR^2^) of the multiple regression models using the HWSD or SoilGrids soil classes as predictors of field‐measured soil cation concentration ranged 0.25–0.32 for the UTU data, 0.38–0.57 for the PPBio data, 0.29–0.42 for the SOTERLAC data, and 0.20–0.23 for the combined data (Table 2). Models based on HWSD had consistently better predictive power than those based on SoilGrids, but including or excluding CEC made little difference.

**Table 1:** Results of Tukey's tests assessing if pairs of dominant soil classes in SOTERLAC differ in mean soil cation concentration in lowland Amazonia. The upper triangle shows the error probabilities for the UTU dataset and the lower triangle for the PPBio dataset. Significant comparisons of soil classes (*p* adj < .001) are shown in bold. Empty cells correspond to dominant soil classes that were not represented in one of the datasets

	Acrisols	Alisols	Cambisols	Ferralsols	Fluvisols	Gleysols	Leptosols	Lixisols	Plinthosols	Podzols
Acrisols	NA	1.000	.638	**.000**	.178	**.000**		.931	**.001**	.308
Alisols		NA	.938	**.000**	.546	.047		.941	.008	.313
Cambisols	**.000**		NA	**.000**	.03	.913		.349	**.000**	.087
Ferralsols	**.000**		**.000**	NA	.094	**.000**		.390	1.000	1.000
Fluvisols					NA	**.000**		1.000	.366	.801
Gleysols						NA		.004	**.000**	.010
Leptosols	.036		**.000**	.978			NA			
Lixisols								NA	.603	.801
Plinthosols	**.000**		**.000**	**.000**			.478		NA	1.000
Podzols	**.000**		**.000**	.005			.966		.342	NA

**Table 2:** Summary of the results of multiple regression models that aim to predict soil cation concentration using the soil class data from either SoilGrids or HWSD. Models were run for UTU, PPBio, and SOTERLAC datasets both separately and combined. In addition, SoilGrids and HWSD are composed of multiple and independent layers that were used as separate variables in the models. The values of soil cation concentration were log‐transformed. The full names of the soil layers are listed in Table A2. AIC = Akaike Information Criterion

Dataset	Soildata	AIC	adjR^2^	*p*‐value
UTU	HWSD	1720	0.31	<.001
	SoilGrids	1795	0.25	<.001
	HWSD + CEC	1720	0.32	<.001
	SoilGrids + CEC	1778	0.27	<.001
PPBio	HWSD	175	0.55	<.001
	SoilGrids	284	0.38	<.001
	HWSD + CEC	167	0.57	<.001
	SoilGrids + CEC	276	0.39	<.001
SOTERLAC	HWSD	521	0.29	<.001
	SoilGrids	515	0.3	<.001
	HWSD + CEC	457	0.42	<.001
	SoilGrids + CEC	474	0.39	<.001
UTU + PPBio + SOTERLAC	HWSD	2899	0.23	<.001
	SoilGrids	2955	0.2	<.001
	HWSD + CEC	2891	0.23	<.001
	SoilGrids + CEC	2919	0.22	<.001

**Figure 3:**
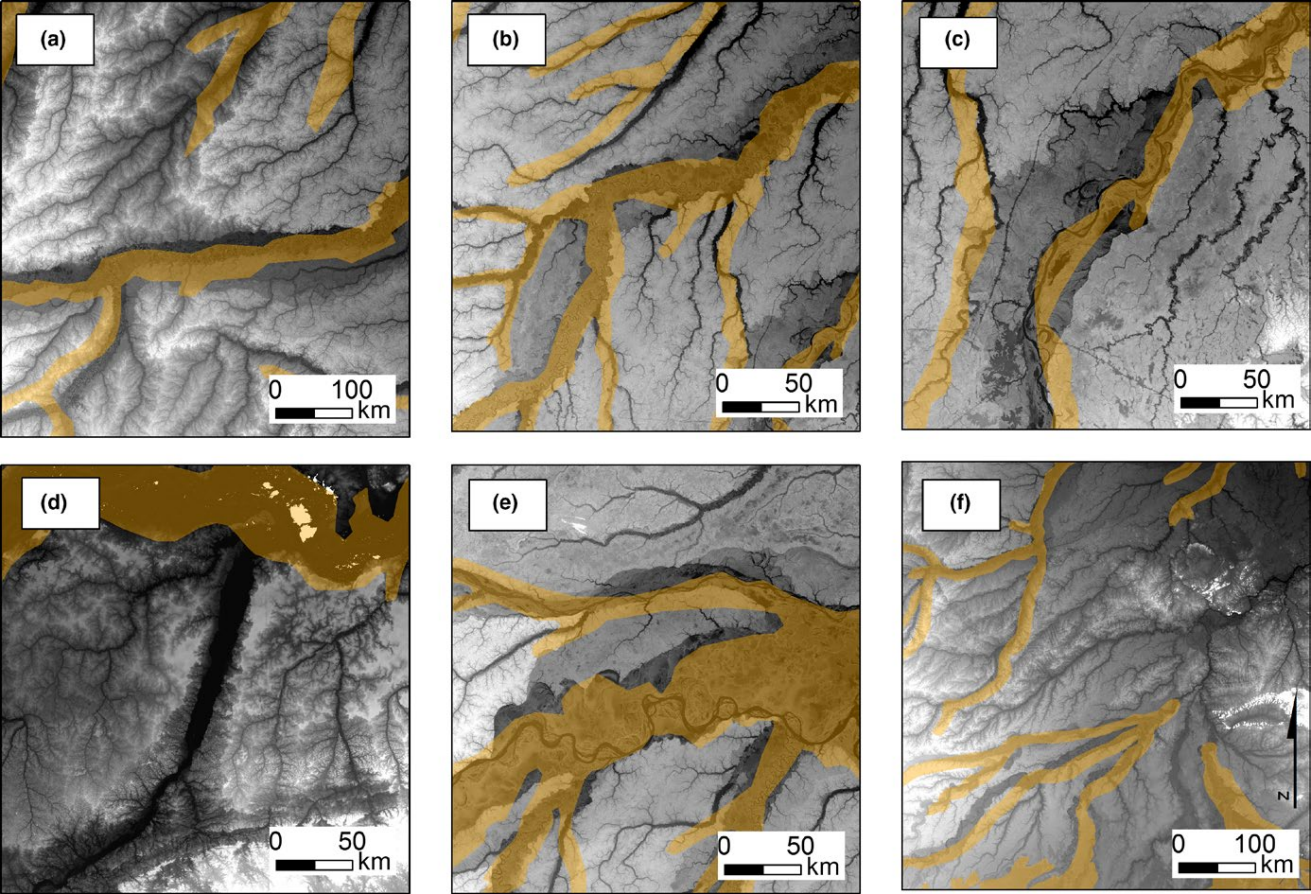
The displacement of Gleysols and Fluvisols, which are typical of inundated areas, in relation to river floodplains. Orange shading shows the distribution of the soil classes as mapped in SOTERLAC, gray background is the SRTM digital elevation model. Dark shades correspond to low elevations (river floodplains and swamps), light shades to high elevations (non‐inundated areas). Details are shown from along six tributaries of the Amazon river: (a) middle Juruá; (b) lower Purus; (c) middle Madeira; (d) lower Tapajós; (e) confluence of the Japurá (North), Solimões (main channel), and Juruá; (f) upper Madeira and upper Purus

**Figure 4:**
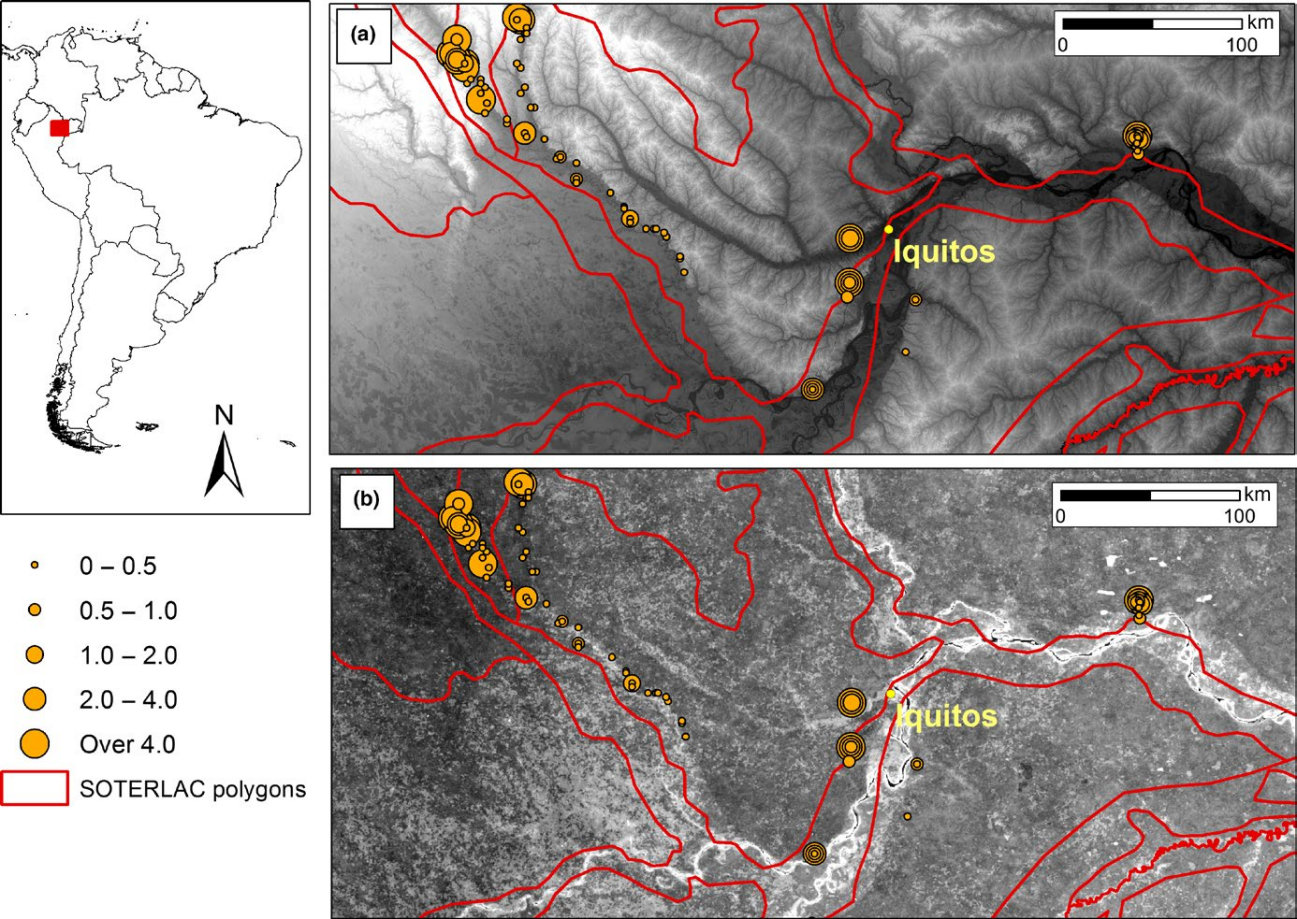
Georeferencing problems in digital soil maps in the Iquitos area, northern Peru: (a) Displacement of SOTERLAC soil class boundaries in relation to the elevational data from SRTM‐DEM. Dark shades correspond to low elevations (river floodplains and swamps), light shades to high elevations (noninundated areas). (b) Soil cation exchange capacity (CEC) values obtained from SoilGrids (lighter shades correspond to higher values) in relation to the SOTERLAC soil class boundaries. Orange dots correspond to soil samples, and their size is proportional to measured soil cation concentration value as shown in the inset (in cmol(+)/kg)

The visual comparison of the SOTERLAC and HWSD soil maps with SRTM elevation data revealed severe georeferencing problems. Soil classes typical of inundated areas (Gleysols, Fluvisols) were displaced by up to 20 km from the river floodplains they were obviously meant to follow, and were instead mapped onto areas that the SRTM shows to be non‐inundated (Figures 3 and 4a). This causes soil samples from these areas to get associated with the wrong soil class in the numerical analyses, which can significantly increase the range of soil cation concentration values associated with the affected soil classes. Although HWSD has a higher nominal resolution than SOTERLAC (1‐km pixel vs. large polygons), it suffers from the same georeferencing problems. In this respect, SoilGrids has corrected these issues (Figure 4b).

Another potential source of inaccuracy is that an area may have more heterogeneous soils than is apparent from the soil maps. We assessed this in the non‐inundated area around Iquitos, Peru, which we know from field experience to contain a mosaic of soil types ranging from extremely poor white sands (Arenosols) to cation‐rich clay soils (Alisols). However, the spatial resolution of the SOTERLAC map is not sufficient to separate these edaphically contrasting patches into different polygons (Figure 4a). Therefore, the SOTERLAC soil classes that are assigned to the large polygons close to Iquitos necessarily receive broad cation concentration ranges. For example, the measured cation concentration in soil samples taken within a single polygon ranged 0.12 – 37.59 cmol/kg for Haplic Acrisols (ACh) and 0.30 – 22.33 cmol/kg for Gleysols (GLe).

### 3.2 Optima and tolerances of taxa along soil gradients

**Figure 5:**
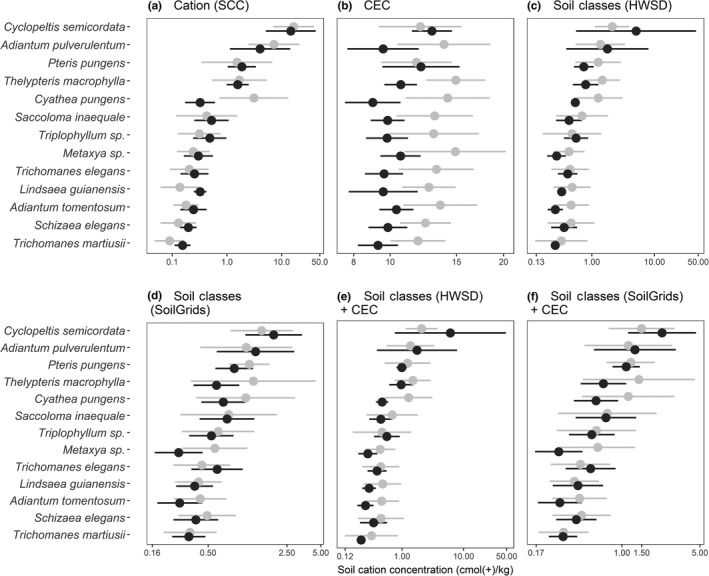
Optima (circles) and tolerances (horizontal bars) of thirteen fern taxa along six different soil gradients as calculated separately for UTU (gray lines) and PPBio (black lines). Soil gradient based on (a) soil cation concentration (SCC) measured from soil samples of the PPBio and UTU datasets; (b) cation exchange capacity (CEC) from SoilGrids; (c‐d) soil cation concentration as estimated from HWSD or SoilGrids soil class data; (e‐f) soil cation concentration as estimated from HWSD or SoilGrids soil class data together with CEC. For the variables used in the regression models, see Appendix 1, Table A2. Taxa are sorted according to the mean of the two optimum values in (a)

When based on the soil cation concentration gradient derived from actual soil samples, the tolerances of the fern taxa were narrow and the taxon optima were well distributed in both the PPBio and UTU datasets. In addition, the rank orders of the taxon optima were almost identical (Figure 5a, Table 3).

**Table 3:** Summary of Kendal's tau rank correlations between the rank orders of species optima along a soil cation concentration gradient as calculated in two different ways. One set of optima was based on soil cation concentrations measured from soil samples (Figure 5a) and the other on soil cation concentrations predicted using each of the regression models shown in Table 2 in turn. The lettering in the column names (B‐F) corresponds to the panels in Figures 5, 6. Analyses were carried out for UTU and PPBio data both separately and combined. p‐values are shown in brackets

	B ‐ CEC	C ‐ Soil Classes (HWSD)	D ‐ Soil Classes (SoilGrids)	E ‐ Soil Classes (HWSD) + CEC	F ‐ Soil Classes (SoilGrids) + CEC
UTU	.23 (0.306)	.67 (0.001)	.82 (0.000)	.72 (0.000)	.79 (0.000)
PPBio	.33 (0.129)	.77 (0.000)	.64 (0.002)	.79 (0.000)	.74 (0.000)
Both	.08 (0.570)	.61 (0.000)	.66 (0.000)	.62 (0.000)	.67 (0.000)

When taxon optima were calculated based on the soil cation concentration gradient predicted using the HWSD and SoilGrids soil class probabilities (Table 3, Figure 5c,d), relatively similar results were obtained than with the actual soil sample data. The rankings of taxon optima based on these two approaches were highly correlated both for the UTU and the PPBio data separately and for the combined dataset (UTU: Kendall's tau = 0.67–0.82, *p* < .001; PPBio: Kendall's tau = 0.64–0.77, *p* < .002; combined: Kendall's tau = 0.61–0.66, *p* < .001;). However, the optima based on predicted soil cation concentration values were less spread out along the gradient than the optima based on measured values. Consequently, the predicted tolerances overlapped more broadly between species than the measured tolerances did.

**Figure 6:**
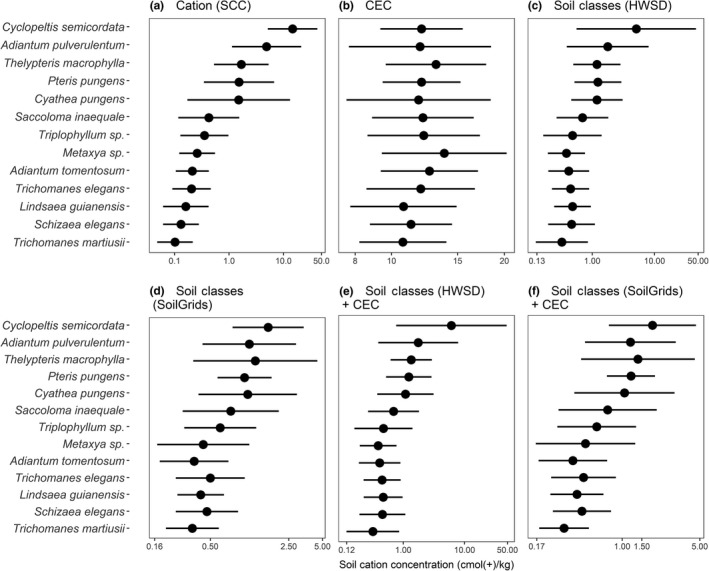
Optima (circles) and tolerances (horizontal bars) of thirteen fern taxa along six different edaphic gradients. The values were combined by taking the minimum and maximum tolerances of each species from PPBio and UTU datasets. (a) Estimated optima and tolerances for measured soil cation concentration (SCC) as obtained by combining floristic and edaphic field data from the PPBio and UTU datasets. (b) Estimated optima and tolerances for cation exchange capacity (CEC) as obtained by combining floristic field data with SoilGrids CEC data. (c‐f) Estimated optima and tolerances for fitted values of soil cation concentration as obtained by combining floristic field data and the best regression model for soil data (see Table 2). Taxa are sorted according to their optimum in (a)

Taxon optima along the CEC gradient derived from SoilGrids lacked consistency between the UTU and PPBio datasets (Figure 5b). Moreover, the tolerances of the individual taxa covered a much larger proportion of the mapped CEC gradient than of either the field‐observed or the predicted soil cation concentration gradient. With few exceptions, the CEC optimum of a given taxon was much lower when calculated using the PPBio dataset than when using the UTU dataset. This reflects the fact that most UTU sites were in western Amazonia, where the mapped CEC values are generally higher than in central Amazonia, where most PPBio sites were. When the UTU and PPBio data were combined, the CEC tolerances of all species covered most of the mapped CEC gradient (Figure 6). The rankings of the taxon optima based on map‐derived CEC values were not correlated with optima based on field‐measured soil cation concentrations for either the UTU or the PPBio data (UTU: Kendall's tau = 0.23, *p* = .306; PPBio: Kendall's tau = 0.33, *p* = .129).

## 4 DISCUSSION

Even though soil properties are known to be important determinants of plant distribution patterns at the local and regional scales in Amazonia, few attempts have been made to use soil data in species distribution modeling at the extent of the entire Amazon basin. Climatic layers have been much more widely used, partly because climate is thought to be more relevant at broad scales, but no doubt also because ecologically relevant climatic data have been easily available in digital GIS formats for some time already (Mod et al., 2016). Although digital soil data covering the entire Amazon basin have recently become available (SOTERLAC, HWSD, and SoilGrids), our results indicate that their limitations have to be considered when they are used to infer species edaphic niches.

Our results confirmed earlier findings that significant differences exist among the thirteen fern taxa in their optima and tolerances along the soil cation concentration gradient (Tuomisto & Poulsen, 1996; Zuquim et al., 2014). Importantly, these results were very consistent across the independent UTU and PPBio datasets, even though the two had applied different field and laboratory protocols and had been collected over a long time period. This suggests that the affinity of a species to a specific level of soil cation concentration is consistent (Tuomisto, 2006; Zuquim et al., 2012).

We found that the soil classes had low to intermediate correspondence with field‐measured soil cation concentrations. Because Amazonia harbors soil classes that vary widely in their pedogenesis as well as in chemical and physical properties (Quesada et al., 2010), we expected that mapped soil class information could be used to infer spatial heterogeneity in such soil properties that would be important in species distribution modeling (SDMs). In particular, we expected that cation‐poor soil classes would clearly differ from cation‐rich soil classes. However, this was not the case, which reduces the usefulness of the soil maps for applications that depend on identifying where edaphically suitable sites for plant species of interest might be found. The low correspondence between the true predictor variable (field data) and the digital environmental layer suggests that the predictions of SDMs based on these data would have high uncertainties (McInerny & Purves, 2011). Our results are related to three main problems in the digital soil maps: (1) insufficient resolution and thematic accuracy, (2) georeferencing problems, and (3) absence of relevant variables. Each of these will be discussed in turn.

### 4.1 Insufficient resolution and thematic accuracy

The international soil science community has invested considerable effort in producing global soil maps, and these are no doubt useful for many purposes (Hartemink et al., 2013). However, it is a recognized problem that the accuracy of soil maps in Amazonia is low (Laurance et al., 2002) due to the limited and fragmented field knowledge about the spatial distribution of different kinds of soils and their properties. This can be problematic for species distribution modeling and other applications that depend on correctly identifying both the edaphic affinities of species and the spatial distribution of the suitable edaphic conditions.

SOTERLAC is available as a vector map, in which resolution is constrained by polygon size. In most of Amazonia, the polygons are very large, in many cases more than 100 km across. Polygons that are larger than the patches of significantly different soils necessarily become internally heterogeneous. The larger the discrepancy between polygon size in the map and the patch size of actual soil heterogeneity in the field, the bigger the problem caused by low spatial resolution. In extreme cases, significant soil variation is not shown in the soil map at all.

Our results showed that differences in cation concentration of up to two orders of magnitude can be found within a single SOTERLAC polygon. For some soil classes, a single outlier soil sample extended the observed soil cation concentration range notably, but in most cases, the measured cation concentration values were well distributed over the range (Figure 2). Nevertheless, in spatial analyses, the polygons have to be treated as if any attribute values were uniform within them, so internal heterogeneity will cause noise and reduce the accuracy of SDMs. The resolution discrepancies can cause soil samples and plant occurrences to become associated with the wrong soil class. As a result, the soil maps may indicate as suitable for a given species such soil classes on which the species in reality does not occur but appears to do so on the basis of the soil map.

HWSD and SoilGrids are available as raster maps, in which spatial resolution depends on pixel size (1 km and 250 m, respectively). In these maps, the spatial resolution can be considered high, but the actual thematic information is unlikely to be accurate at this resolution. Indeed, the SOTERLAC polygon limits are clearly visible in the HWSD, which therefore suffers from partly the same problems. SoilGrids, on the other hand, is based on machine learning algorithms and its thematic resolution can, in principle, be upgraded according to the covariates used in the mapping. However, accuracy is still a challenge, because it is dependent on the availability of local soil information as an input for the mapping.

We found that the relationships between fern taxa and CEC values were inconsistent between the UTU and PPBio datasets. In general, soil heterogeneity is higher in western Amazonia than in central Amazonia (Quesada & Lloyd, 2016; Sombroek, 2000). A very long gradient in soil cation concentration can be found within a few kilometers in western Amazonia (Higgins et al., 2011; Tuomisto & Ruokolainen, 1994), whereas central Amazonia seems to lack the high‐cation soils entirely. These regional differences notwithstanding, our results based on measured soil cation concentration were consistent between the UTU and PPBio datasets. In contrast, our results based on map‐derived CEC were far from consistent. This indicates that predictions made using the mapped CEC values may not reflect local conditions adequately, but might be overly sensitive to assumed continent‐wide trends. Consequently, studies that use CEC as the soil variable in species modeling (e.g., Levis et al., 2017; McMichael et al., 2014) may have underestimated the importance of soils to explain floristic patterns.

### 4.2 Georeferencing problems

A visual comparison of the SOTERLAC map with SRTM topographical data revealed that there are relevant georeferencing errors in some of the limits between soil classes. In particular, along many rivers, the soil classes typical of inundated areas did not coincide with the actual river floodplains, and often the displacement was in the order of 20 km or more. The original SOTERLAC maps were produced at a small scale of 1:1 million or even 1:5 million, and at that scale such errors are marginal. The situation becomes very different when the maps are digitized, because then they can be zoomed in and the digital polygons seem to have exact limits at all scales. However, their real accuracy is no better than that of the original small‐scale map, which will cause problems in GIS analyses that overlay data from different sources on the basis of exact coordinates. The same georeferencing errors are retained in HWSD and the 1‐km resolution version of SoilGrids, which was produced using HWSD as covariate (Hengl et al., 2014). In the newer version of SoilGrids at 250‐m resolution (which was used in our analyses), the displacement of the floodplains has been corrected with the help of the SRTM digital elevation model (Hengl et al., 2017).

Global soil maps can be very useful in providing information about general trends across continents, but their local inaccuracy becomes an issue when they are used in species‐soil assessments. A georeferencing error of just a few hundred meters between contrasting soil classes may be sufficient to create an artefactual association between a taxon and a soil type, which is likely to cause the soil associations of taxa to appear less specialized than they actually are. This, in turn, can have a major impact on both which areas are modeled to contain suitable soils for a taxon of interest, and how large those suitable areas are predicted to be. Errors in such predictions can have serious impacts when the results are used to guide conservation planning or other decisions that have implications for biodiversity. Given that accessibility issues have caused data collecting in Amazonia to become highly concentrated along the rivers (McMichael et al., 2017), the georeferencing problems we identified can be expected to be especially severe.

The usual approach in species distribution modeling is to take the available environmental data layers and accept them at face value, because analysts rarely have the possibility to do otherwise. Species modeling techniques allow using both vector maps and raster maps simultaneously. Raster maps usually provide quantitative information, while vector maps are more often associated with qualitative information. Identifying errors requires detailed scrutiny of the data against another data source or field knowledge, and even if problems are identified, correcting them can be a daunting task (the more so the bigger the area of interest) (Hengl et al., 2017). Georeferencing errors related to the limits of floodplains and their associated soil classes can, in principle, be identified and corrected using a high‐resolution map of Amazonian wetlands (Hess et al., 2015). However, limits between soil types in the vast non‐inundated areas are more difficult to detect and correct. Species distribution models therefore need to allow for large locational errors to diminish the effect of georeferencing problems associated with the maps, which in turn may reduce their thematic accuracy.

### 4.3 Absence of relevant variables

We found the correlation between measured soil cation concentration and mapped CEC to be very low. Many ecological studies have shown that soil cation concentration (specifically, the concentration of the base cations Ca, Mg, and K) is among the most important variables to explain plant species occurrence patterns in Amazonia (Pansonato, Costa, de Castilho, Carvalho, & Zuquim, 2013; Phillips et al., 2003; Tuomisto, Ruokolainen, & Yli‐Halla, 2003; Tuomisto et al., 2016; Zuquim et al., 2014). However, this variable is not provided in any of the currently available digital soil maps. SoilGrids provides CEC (cation exchange capacity), which is related to cations but has problems as a surrogate measure: It quantifies the potential of the soil to bind cations in general (including aluminum), not the concentration of base cations that are actually present in the soil and available to plants. For example, the Soilgrids CEC fails to reflect a 1,000‐km‐long limit between geological formations that is associated with contrasting soils, vegetation, and plant species composition at the border between western and central Amazonia (Higgins et al., 2011; IBGE 2004; Tuomisto et al., 2016).

The number of soil classes has sometimes been used as an indicator of soil heterogeneity, and CEC has been used as an indicator of soil fertility, but these variables have not been found significant in species distribution and diversity assessments (Kissling et al., 2012; McPherson, 2014). In our analyses, the ranking of fern taxa by their cation concentration optima could, to some degree, be reconstructed using a combination of soil class data from HWSD and CEC data from SoilGrids. On the other hand, species tolerances had low correspondence with the estimate tolerances based on field data in these analyses. As the regional differences in CEC values seemed to be excessive and the HWSD suffered from georeferencing issues, these results are probably very sensitive to the exact geographic positions of the sampling points. Soil classification data based on the WRB‐FAO system are available in all three digital soil maps, but this classification does not necessarily reflect those soil properties that are physiologically most relevant for plant species (Grunwald et al., 2011; Lips & Duivenvoorden, 1996; Sollins, 1998).

### 4.4 Perspectives on soil mapping in Amazonia

Our results showed that species edaphic affinites for soil cation concentration had low correspondence when derived using data from soil samples versus soil class information from soil maps. Although the rank orders were similar for optima derived from map data versus field data, the actual positions of the optima were more similar for the map‐based data and also species tolerances were broader. This suggests that predictions based on single data layers will probably overestimate the suitable areas for species occurrence. However, regression models that used several layers from the soil maps simultaneously gave better results, and might provide an approach to extracting more useful environmental data for SDMs.

Ideally, soil maps themselves will gradually become more accurate. A critical point here is that more validation points are needed. Initiatives such as the World Soil Information System (WoSIS, Batjes et al., 2017) and the Global Soil Information Facilities (GSIF, http://www.isric.org/explore/gsif) are therefore welcomed. These encourage the establishment of open databases with standardized sampling and laboratory methods for measuring soil properties. The new validation points can then be used to update the soil maps (Hengl et al., 2017). In addition, covariates are of key importance to improve map resolution and accuracy, especially in areas where no validation points exist. The SRTM topography data have already been used to improve the accuracy of SoilGrids, and new products from earth observation satellites and other remotely sensed data may provide further improvements.

## 5 CONCLUSIONS

We found that even when field data show Amazonian plant taxa to have highly specific soil cation concentration associations, it is difficult to reconstruct these using the information contained in currently available digital soil maps (SOTERLAC, HWSD, SoilGrids). None of these provides data on soil cation concentration or other soil properties that have been found important for plant species distributions in ecological studies. The ranking of species' soil cation concentration optima was poorly reconstructed by optima based on the cation exchange capacity (CEC) values available in SoilGrids. Regression models based on the soil class information available in HWSD and SoilGrids succeeded better, but even here the species tolerances overlapped more than those based on field data, causing the species to appear less segregated in their edaphic niches than they are according to field data. The SOTERLAC and HWSD maps suffer from major georeferencing errors, but these have been corrected in the new version of SoilGrids at 250‐m resolution. Overall, our analyses indicated that soil maps for Amazonia still need to be improved in order to provide better data layers for the assessment of species–soil associations and for species distribution modeling.

## CONFLICT OF INTEREST

None declared.

## AUTHOR CONTRIBUTIONS

GM, GZ, and HT conceived the original idea; all authors collected data; GM performed the analyses; GM, GZ, and HT wrote the manuscript with contributions from all the others.

## DATA ACCESSIBILITY

Data available from the Dryad Digital Repository: https://doi.org/10.5061/dryad.jm00k.
