# Supplementary material for: Using digital soil maps to infer edaphic affinities of plant species in Amazonia: Problems and prospects
Source: Ecol Evol. 2017 Sep 12;7(20):8463–77. doi: 10.1002/ece3.3242 (PMC5648677; doi:10.1002/ece3.3242)
Supplement: Supplementary file 1 [file ECE3-7-8463-s001.docx]

**Appendix 1**

Moulatlet, G.M. et al. Using digital soil maps to infer edaphic affinities of plant species in Amazonia: problems and prospects

Table A1. Summary of soil information. Soil classes as extracted from SOTERLAC map and their full name. Mean values and ranges (in brackets) are shown in cmol(+)/kg for each of the soil datasets.

| Soil Class |  | SOTERLAC | UTU | PPBio |
| --- | --- | --- | --- | --- |
| ACf | Ferric Acrisols | 2.39 ( 0.47 - 8.74 ) | NA ( NA - NA ) | NA ( NA - NA ) |
| ACh | Haplic Acrisols | 5.55 ( 0.12 - 37.59 ) | 3.46 ( 0.05 - 53.59 ) | 2.33 ( 0.24 - 9.07 ) |
| ACp | Plinthic Acrisols | 1.86 ( 0.41 - 5.66 ) | NA ( NA - NA ) | NA ( NA - NA ) |
| ACu | Humic Acrisols | 0.23 ( 0.23 - 0.23 ) | NA ( NA - NA ) | NA ( NA - NA ) |
| ALu | Humic Alisols | NA ( NA - NA ) | 4.32 ( 0.08 - 25.56 ) | NA ( NA - NA ) |
| ARg | Gleyic Arenosols | 0.2 ( 0.15 - 0.26 ) | NA ( NA - NA ) | NA ( NA - NA ) |
| ARh | Haplic Arenosols | 6.56 ( 0.12 - 13 ) | NA ( NA - NA ) | NA ( NA - NA ) |
| CMd | Dystric Cambisols | 10.72 ( 0.23 - 67.1 ) | 14.05 ( 0.17 - 38.92 ) | NA ( NA - NA ) |
| CMe | Eutric Cambisols | 10.19 ( 1.94 - 34.36 ) | NA ( NA - NA ) | 28.3 ( 16.81 - 37.09 ) |
| CMg | Gleyic Cambisols | 1.94 ( 1.94 - 1.94 ) | NA ( NA - NA ) | NA ( NA - NA ) |
| CMo | Ferralic Cambisols | NA ( NA - NA ) | 2.65 ( 0.11 - 27.09 ) | NA ( NA - NA ) |
| FLd | Dystric Fluvisols | 1.64 ( 0.8 - 2.48 ) | NA ( NA - NA ) | NA ( NA - NA ) |
| FLe | Eutric Fluvisols | 10.25 ( 1.39 - 25.22 ) | 1.63 ( 0.1 - 18.91 ) | NA ( NA - NA ) |
| FRh | Haplic Ferralsols | 3.22 ( 0.12 - 16.47 ) | NA ( NA - NA ) | 0.99 ( 0.2 - 5.94 ) |
| FRr | Rhodic Ferralsols | 0.48 ( 0.27 - 0.75 ) | NA ( NA - NA ) | NA ( NA - NA ) |
| FRu | Humic Ferralsols | 0.46 ( 0.18 - 0.97 ) | 0.12 ( 0.03 - 0.31 ) | 0.46 ( 0.23 - 1.04 ) |
| FRx | Xanthic Ferralsols | 1.61 ( 0.13 - 21.03 ) | 0.68 ( 0.12 - 5.53 ) | 0.85 ( 0.1 - 5.68 ) |
| GLd | Dystric Gleysols | 1.22 ( 0.32 - 2.86 ) | 0.31 ( 0.05 - 2.12 ) | NA ( NA - NA ) |
| GLe | Eutric Gleysols | 8.32 ( 0.3 - 22.33 ) | 7.99 ( 0.06 - 33.95 ) | NA ( NA - NA ) |
| LPd | Dystric Leptosols | 1.54 ( 0.11 - 9.75 ) | NA ( NA - NA ) | 0.46 ( 0.26 - 0.77 ) |
| LPe | Eutric Leptosols | 11.71 ( 2.48 - 34.15 ) | NA ( NA - NA ) | NA ( NA - NA ) |
| LPu | Umbric Leptosols | 20.56 ( 0.96 - 47.78 ) | NA ( NA - NA ) | NA ( NA - NA ) |
| LXf | Ferric Lixisols | 26.69 ( 12.64 - 40.74 ) | NA ( NA - NA ) | NA ( NA - NA ) |
| LXh | Haplic Lixisols | 5.97 ( 0.24 - 22.53 ) | 1.25 ( 0.15 - 12.64 ) | NA ( NA - NA ) |
| NTr | Rhodic Nitisols | 27.8 ( 27.8 - 27.8 ) | NA ( NA - NA ) | NA ( NA - NA ) |
| PTa | Albic Plinthosols | 1.4 ( 0.18 - 6.74 ) | NA ( NA - NA ) | NA ( NA - NA ) |
| PTd | Dystric Plinthosols | 1.08 ( 0.14 - 5.39 ) | 0.99 ( 0.08 - 14.99 ) | 0.27 ( 0.08 - 1.19 ) |
| PZc | Carbic Podzols | 0.18 ( 0.06 - 0.28 ) | 0.16 ( 0.09 - 0.25 ) | 0.36 ( 0.16 - 0.98 ) |
| PZh | Haplic Podzols | 0.92 ( 0.13 - 3.45 ) | NA ( NA - NA ) | NA ( NA - NA ) |
| RGd | Dystric Regosols | 2.13 ( 0.27 - 5.13 ) | NA ( NA - NA ) | NA ( NA - NA ) |
| RGe | Eutric Regosols | 7.77 ( 1 - 14.5 ) | NA ( NA - NA ) | NA ( NA - NA ) |
| RGu | Umbric Regosols | 0.21 ( 0.21 - 0.21 ) | NA ( NA - NA ) | NA ( NA - NA ) |
| SCn | Sodic Solonchacks | 4.09 ( 0.45 - 13.48 ) | NA ( NA - NA ) | NA ( NA - NA ) |

Table A2. List of variables used in the multiple regression models.

| Soil map | Layer code | Source | Code Used in the Analysis |
| --- | --- | --- | --- |
| HWSD | GPZHWS3 - Percent coverage Podzols | http://www.worldgrids.org/doku.php/wiki:layers | GPZHWS3 |
| HWSD | GPTHWS3 - Percent coverage Plinthosols | http://www.worldgrids.org/doku.php/wiki:layers | GPTHWS3 |
| HWSD | GLXHWS3 - Percent coverage Lixisols | http://www.worldgrids.org/doku.php/wiki:layers | GLXHWS3 |
| HWSD | GLPHWS3 - Percent coverage Leptosols | http://www.worldgrids.org/doku.php/wiki:layers | GLPHWS3 |
| HWSD | GGLHWS3 - Percent coverage Gleysols | http://www.worldgrids.org/doku.php/wiki:layers | GGLHWS3 |
| HWSD | GFRHWS3 - Percent coverage Ferralsols | http://www.worldgrids.org/doku.php/wiki:layers | GFRHWS3 |
| HWSD | GFLHWS3 - Percent coverage Fluvisols | http://www.worldgrids.org/doku.php/wiki:layers | GFLHWS3 |
| HWSD | GCMHWS3 - Percent coverage Cambisols | http://www.worldgrids.org/doku.php/wiki:layers | GCMHWS3 |
| HWSD | GARHWS3 - Percent coverage Arenosols | http://www.worldgrids.org/doku.php/wiki:layers | GARHWS3 |
| HWSD | GALHWS3 - Percent coverage Alisols | http://www.worldgrids.org/doku.php/wiki:layers | GALHWS3 |
| HWSD | GACHWS3 - Percent coverage Acrisols | http://www.worldgrids.org/doku.php/wiki:layers | GACHWS3 |
| SoilGrids | TAXNWRB_Ferralic.Cambisols_250m_ll.tif | ftp://ftp.soilgrids.org/data/recent/ | CMf |
| SoilGrids | TAXNWRB_Haplic.Acrisols..Ferric._250m_ll.tif | ftp://ftp.soilgrids.org/data/recent/ | hACf |
| SoilGrids | TAXNWRB_Haplic.Acrisols_250m_ll.tif | ftp://ftp.soilgrids.org/data/recent/ | ACh |
| SoilGrids | TAXNWRB_Haplic.Alisols_250m_ll.tif | ftp://ftp.soilgrids.org/data/recent/ | ALh |
| SoilGrids | TAXNWRB_Haplic.Arenosols_250m_ll.tif | ftp://ftp.soilgrids.org/data/recent/ | ARh |
| SoilGrids | TAXNWRB_Haplic.Cambisols..Dystric._250m_ll.tif | ftp://ftp.soilgrids.org/data/recent/ | hCMd |
| SoilGrids | TAXNWRB_Haplic.Cambisols..Humic._250m_ll.tif | ftp://ftp.soilgrids.org/data/recent/ | hCMu |
| SoilGrids | TAXNWRB_Haplic.Cambisols_250m_ll.tif | ftp://ftp.soilgrids.org/data/recent/ | CMh |
| SoilGrids | TAXNWRB_Haplic.Ferralsols..Xanthic._250m_ll.tif | ftp://ftp.soilgrids.org/data/recent/ | hFRx |
| SoilGrids | TAXNWRB_Haplic.Ferralsols_250m_ll.tif | ftp://ftp.soilgrids.org/data/recent/ | FRh |
| SoilGrids | TAXNWRB_Haplic.Fluvisols..Eutric._250m_ll.tif | ftp://ftp.soilgrids.org/data/recent/ | hFLe |
| SoilGrids | TAXNWRB_Haplic.Fluvisols_250m_ll.tif | ftp://ftp.soilgrids.org/data/recent/ | FLh |
| SoilGrids | TAXNWRB_Haplic.Gleysols..Dystric._250m_ll.tif | ftp://ftp.soilgrids.org/data/recent/ | hGLd |
| SoilGrids | TAXNWRB_Haplic.Gleysols..Eutric._250m_ll.tif | ftp://ftp.soilgrids.org/data/recent/ | hGLe |
| SoilGrids | TAXNWRB_Haplic.Gleysols_250m_ll.tif | ftp://ftp.soilgrids.org/data/recent/ | GLh |
| SoilGrids | TAXNWRB_Haplic.Leptosols_250m_ll.tif | ftp://ftp.soilgrids.org/data/recent/ | LPh |
| SoilGrids | TAXNWRB_Haplic.Lixisols_250m_ll.tif | ftp://ftp.soilgrids.org/data/recent/ | LXh |
| SoilGrids | TAXNWRB_Haplic.Podzols_250m_ll.tif | ftp://ftp.soilgrids.org/data/recent/ | PZh |
| SoilGrids | TAXNWRB_Plinthic.Acrisols_250m_ll.tif | ftp://ftp.soilgrids.org/data/recent/ | ACp |
| SoilGrids | CECSOL_M_sl2_250m_ll.tif | ftp://ftp.soilgrids.org/data/recent/ | CEC |
